# DNA Display II. Genetic Manipulation of Combinatorial Chemistry Libraries for Small-Molecule Evolution

**DOI:** 10.1371/journal.pbio.0020174

**Published:** 2004-06-22

**Authors:** David R Halpin, Pehr B Harbury

**Affiliations:** **1**Department of Biochemistry, Stanford University School of MedicineStanford, CaliforniaUnited States of America

## Abstract

Biological in vitro selection techniques, such as RNA aptamer methods and mRNA display, have proven to be powerful approaches for engineering molecules with novel functions. These techniques are based on iterative amplification of biopolymer libraries, interposed by selection for a desired functional property. Rare, promising compounds are enriched over multiple generations of a constantly replicating molecular population, and subsequently identified. The restriction of such methods to DNA, RNA, and polypeptides precludes their use for small-molecule discovery. To overcome this limitation, we have directed the synthesis of combinatorial chemistry libraries with DNA “genes,” making possible iterative amplification of a nonbiological molecular species. By differential hybridization during the course of a traditional split-and-pool combinatorial synthesis, the DNA sequence of each gene is read out and translated into a unique small-molecule structure. This “chemical translation” provides practical access to synthetic compound populations 1 million-fold more complex than state-of-the-art combinatorial libraries. We carried out an in vitro selection experiment (iterated chemical translation, selection, and amplification) on a library of 10^6^ nonnatural peptides. The library converged over three generations to a high-affinity protein ligand. The ability to genetically encode diverse classes of synthetic transformations enables the in vitro selection and potential evolution of an essentially limitless collection of compound families, opening new avenues to drug discovery, catalyst design, and the development of a materials science “biology.”

## Abstract 2

The authors use DNA "genes" to direct the synthesis of combinatorial chemistry libraries, and show in an in vitro selection experiment that specific drugs can be developed

## Introduction

Creation of molecular function represents a fundamental challenge. Nature accomplishes the task through evolution, iterating cycles of selection, amplification, and diversification. Multiple generations of selective pressure and reproduction transform a diverse population into one consisting only of molecules fit to survive. Life on this planet thus emerged from a limited chemical palette, comprising proteins, nucleic acids, sugars, lipids, and metabolites. Over the last two decades, technologies that recapitulate this process in the test tube have been developed, and have produced an amazing collection of biopolymers with unprecedented recognition and catalytic properties (reviewed in Roberts and Ja 1999). At present, however, these in vitro selection techniques cannot be applied to compounds of nonbiological origin and have therefore not affected most areas of molecular discovery. The question arises: what would become possible if in vitro selection were applied to chemical populations of arbitrary composition?

High-throughput screening of combinatorial chemistry (HTS-CC) libraries represents a first approximation to small-molecule evolution, in that the process roughly mimics the diversification and selection components of evolution. However, amplification and iteration have no functional equivalents in HTS-CC techniques, placing practical limits on library complexity. Amplification and iteration are critical for identifying vanishingly small amounts of material from a complex population. Moreover, these processes make possible the application of bulk selections rather than serial screens to assay libraries, vastly increasing throughput. Accordingly, typical HTS-CC libraries rarely exceed 10^6^ unique members (Dolle 2003), whereas the biopolymer libraries used for in vitro selection experiments generally comprise 10^9^–10^13^ unique members (Roberts and Ja 1999). A state-of-the-art high-throughput screening facility, capable of performing 300,000 tests per day, would require 9 millennia to screen a typical in vitro selection library (Morais 2003). If the success of molecular discovery correlates with library complexity, then in vitro selection of combinatorial chemistry libraries for functional molecules will be far more powerful than screening.

In order to apply in vitro selection to combinatorial chemistry libraries, each compound must be associated with a gene that specifies its structure. DNA has been utilized previously to record the synthetic history of individual beads in a split-and-pool combinatorial synthesis, but the DNA tags could not direct subsequent resynthesis of the corresponding compound (Brenner and Lerner 1992). More recently, hybridization-induced proximity strategies for DNA-templated organic synthesis have been described, but their use has not yet been reported for the synthesis of complex libraries (Gartner and Liu 2001 and references therein). In this manuscript we present and demonstrate a general method for the in vitro selection and evolution of combinatorial chemistry libraries (Harbury and Halpin 2000).

## Results

### Strategy

**Figure 1:**
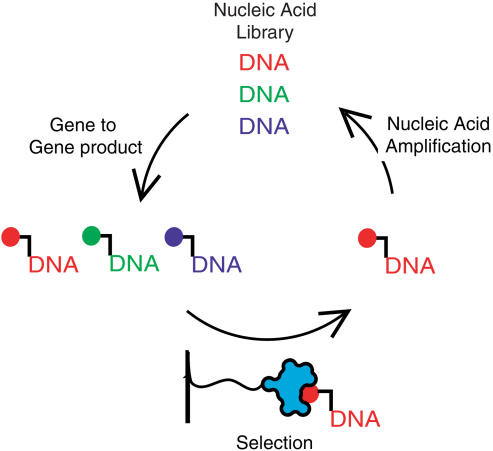
The In Vitro Selection Cycle Experiments are initiated with a nucleic acid library (colored DNA). The sequence of each gene directs the synthesis of a corresponding gene product (colored ball) that is physically linked to its encoding nucleic acid. The gene products are subjected to selection, for example, through binding to an immobilized macromolecule (cyan widget at bottom). The nucleic acid encoding selected gene products is amplified and used as input for a subsequent cycle.

In vitro selection requires iterated rounds of three steps: conversion of genes to gene products, selection of gene products, and gene amplification (Figure 1). The last two steps, selection and amplification, are similar between all forms of in vitro selection. However, conversion of genes to gene products poses a unique problem for the in vitro selection of small molecules. Whereas enzymes convert genetic material into the natural biopolymers, no machinery exists to directly translate genes into small molecules.

**Figure 2:**
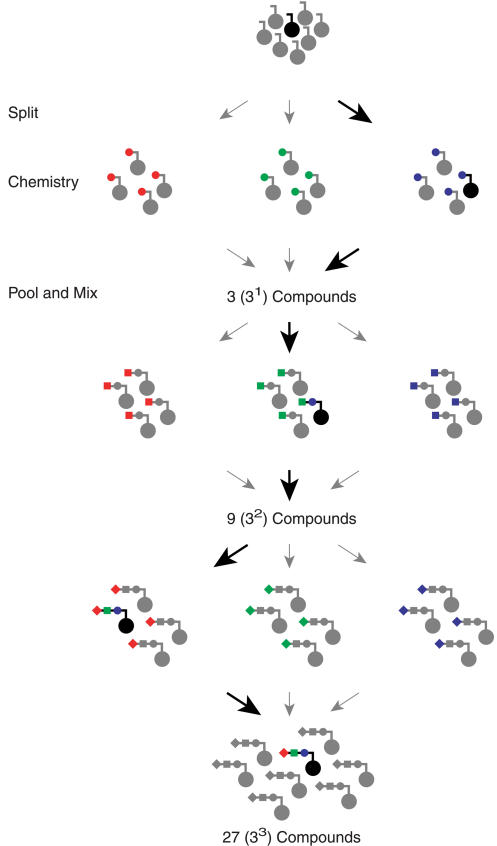
Split-and-Pool Synthesis of a Combinatorial Chemistry Library A mixture of solid supports (balls with rotated “L” at top) is randomly split into subpools. A distinct chemical building block (red, green, or blue ball) is coupled to the supports in each subpool. The supports are repooled and mixed. This process of splitting, chemistry, and pooling is iterated until the library synthesis is complete. The small molecules ultimately synthesized are combinations of the different building blocks (colored circles, squares, and diamonds). As highlighted by the black bead, the path taken by a support through the split-and-pool synthesis (right, middle, left) determines the small molecule synthesized on it (blue ball, green square, red diamond). The number of reactions performed is the sum of the number of subpools in each split (3 + 3 + 3 = 9). The number of unique small molecules generated is the product of the number of subpools in each split (3 × 3 × 3 = 27).

In general, small-molecule libraries are synthesized by the split-and-pool method which is illustrated in Figure 2 (Furka et al. 1991; Thompson and Ellman 1996). A mixture of supports (the inert material on which small molecules are built, typically polystyrene beads) is randomly split into subpools. A distinct chemical building block is then coupled to the supports in each subpool, after which the supports are pooled together and mixed. Splitting, coupling, and pooling are repeated until the library synthesis is complete. The series of subpools into which a support partitions determines what chemical building blocks are added to the support. Thus, the trajectory that a support takes through a split-and-pool synthesis is essentially a molecular recipe. If a support could predetermine its own trajectory, it would encode the synthesis of the small molecule ultimately attached to it. Predetermining support trajectories can be accomplished by using a DNA library as the support material, and by directing the splits through hybridization. The DNA sequence of each support then governs its subpool path, and acts as a genetic blueprint for a small molecule.

**Figure 3:**
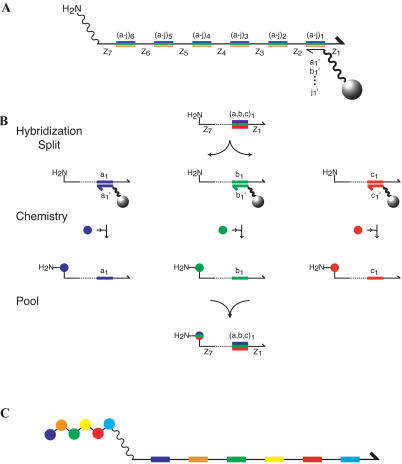
Chemical Translation (A) Schematic showing the structure of the DNA support library. Small molecules are synthesized at the 5′ end of 340-base ssDNA genes. The ssDNA consists of 20-base noncoding regions (black lines labeled Z_1_–Z_7_) and 20-base coding positions (colored bars labeled [a–j]_1–6_). All library members contain the same seven DNA sequences at the seven noncoding regions. At each of the six coding positions, ten mutually exclusive DNA codons, (a–j)_n_, are present, for a total of 60 different sequences. Each coding region specifies the addition of a single subunit to a growing small molecule. A unique reactive site (in this case a primary amine) for small-molecule synthesis is attached to the 5′ end of the ssDNA through a polyethylene glycol linker (squiggly line). Resin beads coated with an oligonucleotide complementary to one codon (anticodon beads, gray ball at right) capture by hybridization ssDNAs containing the corresponding codon. (B) Chemical translation is a split-and-pool synthesis, with splitting directed by DNA hybridization. A ssDNA library is hybridized to a set of anticodon columns (gray balls) corresponding to the set of codons present at a single coding position. The ssDNA genes partition into subpools based on sequence identity. Distinct chemical subunits (colored balls) are coupled to the DNA in each subpool. Finally, the DNA is repooled, completing the encoded addition of one subunit to the growing small molecule. The process of hybridization splitting, chemistry, and pooling is repeated for all subsequent coding regions. (C) Schematic product of chemical translation. The sequence of the small-molecule subunits (colored balls) corresponds to the sequence of codons (colored bars) in the ssDNA gene.

The construct we chose for our DNA support library is shown in Figure 3A. The single-stranded DNA (ssDNA) includes a unique reactive site at its 5′ end, upon which a small molecule is synthesized. The DNA sequence contains 20-base “codons” flanked by 20-base noncoding regions. Within the DNA support library, sequence degeneracy exists at the coding positions. The set of codons in each DNA support specifies a small-molecule synthesis by directing the splitting of the ssDNA into appropriate subpools. The noncoding regions enable genetic recombination of support sequences by PCR (Halpin and Harbury 2004).

Our scheme for DNA-directed split and pool synthesis is shown in Figure 3B. The library is first split by hybridization to a set of anticodon columns complementary to the different 20-base sequences present at the first coding position. Distinct chemical building blocks are coupled to each subpool, and the library is repooled. The process is repeated, but the splitting is directed by a subsequent coding position. Each coding position comprises a set of codons that differ in sequence from the codons at all other coding positions. Consequently, splitting is always directed by hybridization at one intended coding region, and not by codons elsewhere. Small molecules are synthesized directly on their encoding DNAs, maintaining the physical linkage between gene and gene product (Figure 3C). Direct conversion of genes into small-molecule gene products, combined with selection and amplification steps, enables the in vitro selection of small-molecule libraries.

### Reduction to Practice

**Figure 4:**
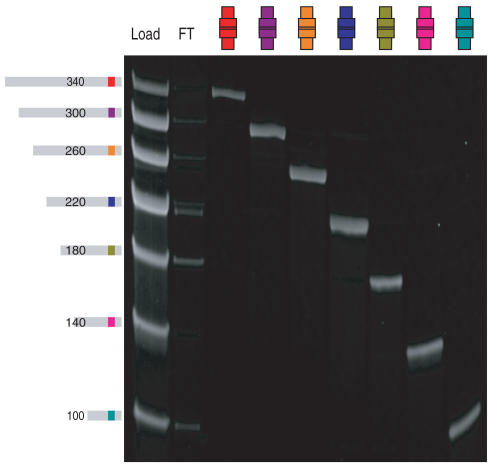
Sequence-Directed Splitting Seven serially truncated ssDNAs differing in sequence at one coding position (illustrated at left of gel, number of bases indicated) were hybridized to seven anticodon columns (cylinders at top of gel). The load (lane 1), flow through (lane 2), and column elutes (lanes 3–9) were analyzed by denaturing polyacrylamide gel electrophoresis.

We first developed a Sepharose-based resin derivatized with anticodon oligonucleotides complementary to codon sequences (Halpin and Harbury 2004). We tested the resin by hybridizing a library consisting of seven ssDNA sequences to a corresponding set of seven different anticodon columns (Figure 4). There was little crosshybridization, which ensures that DNA genes will be accurately translated. Analysis of splitting efficiencies by a scintillation counting assay of radiolabeled ssDNA showed that 90% or more of the ssDNA inputs were recovered from the correct hybridization columns for all tested sequences (Halpin and Harbury 2004). The resin is also robust. We have not observed any loss in efficiency with over 30 cycles of hybridization and elution.

We next addressed chemical synthesis on unprotected DNA. Use of a solid phase in small-molecule synthesis allows for the application of excess reagents, to drive reactions to completion, and simplifies product purification (Merrifield 1963). To realize these advantages, we carried out synthetic steps while DNA was noncovalently bound to diethylaminoethyl (DEAE) Sepharose resin (Halpin et al. 2004). DEAE Sepharose was chosen for solid-phase synthesis because it adsorbs DNA reversibly and in a sequence-independent manner and because it behaves well in organic solvents. Incubation of immobilized DNA with the appropriate reagents results in addition of a building block, completing one step in the synthesis of a small molecule. Following the chemical step, DNA is eluted from the solid phase and manipulated in solution.

**Figure 5:**
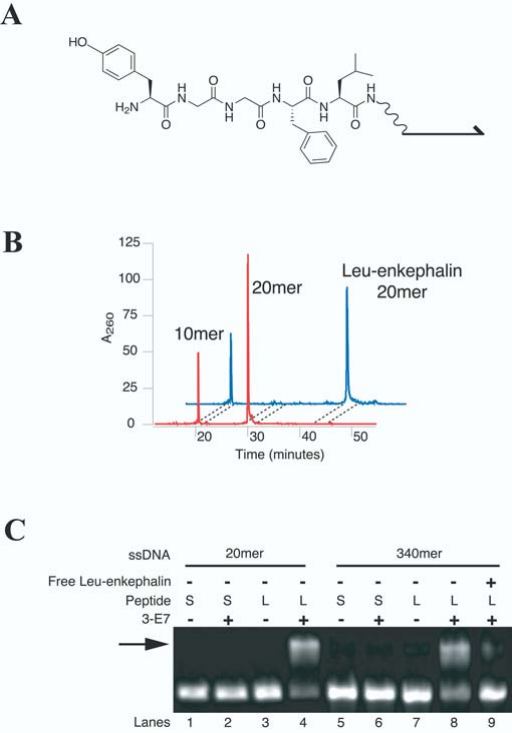
Peptide Synthesis on DNA (A) Structure of the [Leu]enkephalin–DNA conjugate. (B) High performance liquid chromatography chromatogram of the [Leu]enkephalin peptide synthesized using succinimidyl ester chemistry on a 20-base oligonucleotide modified with a 5′ primary amine (20mer). A 10-base oligonucleotide without the 5′ primary amine (10mer) was included in the reactions as a control for nonspecific DNA modification. The red and blue traces are the DNA before and after chemistry, respectively. The mass of the major product peak (42-min retention time) matches the expected mass of the [Leu]enkephalin–DNA conjugate. (C) Electromobility shift assay of peptides synthesized on 340-base ssDNA. Conjugates were eletrophoresed on a native agarose gel in the absence (lanes 1, 3, 5, and 7) or presence (lanes 2, 4, 6, 8, and 9) of the [Leu]enkephalin-binding antibody 3-E7. [Leu]enkephalin (L) or a scrambled sequence (S) was synthesized on a 5′ amino-modified 20-base oligonucleotide, which was subsequently used as a primer for PCR (lanes 1–4), or directly on 5′ amino-modified 340-base ssDNA, which was subsequently converted to dsDNA (lanes 5–9). Addition of free [Leu]enkephalin peptide (lane 9) competes away binding.

As an initial chemistry, we chose 9-Fluorenylmethoxycarbonyl [Fmoc]–based peptide synthesis. Figure 5 shows the results of solid-phase peptide synthesis on DNA using Fmoc-protected succinimidyl esters (Anderson et al. 1963; Carpino and Han 1970; Halpin et al. 2004). Synthesis of the [Leu]enkephalin pentapeptide on an aminated 20-base oligonucleotide (Figure 5B) yielded a highly pure [Leu]enkephalin-DNA conjugate. A nonaminated oligonucleotide internal control was not altered by the chemistry, ruling out nonspecific chemical modification of DNA. Over 90% of the recovered nucleic acid was the intended [Leu]enkephalin-DNA conjugate (the overall recovered yield was 60%). The results correspond to a 98% efficiency for each amino acid coupling step.

Synthesis of [Leu]enkephalin on a 340-base ssDNA support, capable of encoding an eight-step synthesis, was analyzed using an electromobility shift assay and the enkephalin-specific 3-E7 antibody (Hwang et al. 1999). Figure 5C shows that 3-E7 shifts the majority of the [Leu]enkephalin-DNA (approximately 85% when standardized to a positive control), showing that the biological activity of the peptide is maintained while attached to DNA. The 3-E7 antibody does not shift a scrambled-DNA peptide conjugate containing the same amino acids as [Leu]enkephalin but in a different order. Finally, free [Leu]enkephalin peptide eliminates the shifting of [Leu]enkephalin-DNA by 3-E7, demonstrating the specificity of the shift.

**Figure 6:**
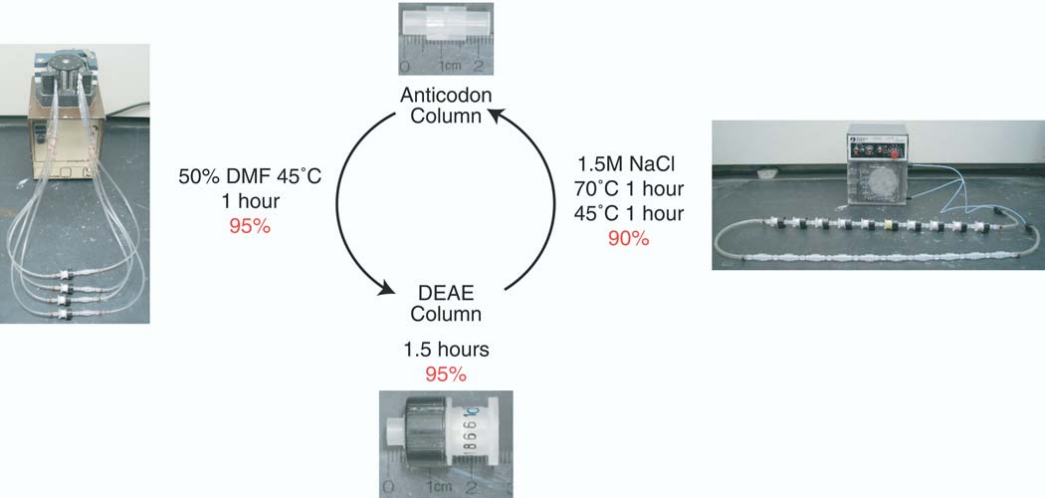
Reduction to Practice Chemical translation requires iteration of a chemistry step and two column-transfer steps. ssDNA is transferred from anticodon columns to DEAE Sepharose columns by cyclically pumping 50% DMF through a pair of columns (one hybridization, one DEAE) attached in series for 1 h at 45 °C. Chemistry is performed on ssDNA bound to each DEAE column. ssDNA is transferred from DEAE columns to anticodon columns by cyclically pumping a 1.5-M NaCl buffer through all DEAE columns and all anticodon columns associated with the next coding position for 1 h at 70 °C and 1 h at 46 °C. Efficiencies for each step are indicated in red.

Our chemical translation strategy requires repeated hybridization-directed splitting and coupling of chemical building blocks to DNA. Two different solid phases were utilized for these tasks. To efficiently transfer DNA from anticodon columns to DEAE Sepharose columns, we cyclically pumped 50% dimethylformamide (DMF) over the columns connected in series (Figure 6). Conversely, to transfer DNA from DEAE Sepharose columns back to anticodon columns, we used a high salt buffer in a closed system. In both cases, a large effective buffer volume flows over each column, which allows the DNA transfer processes to approach thermodynamic equilibrium. These column-to-column transfers remove intermediate storage tubes and require little solvent, minimizing loss of DNA.

### In Vitro Selection of a Chemically Synthesized Library

To test and validate our general strategy, we applied in vitro selection to a primarily nonnatural peptide library, with the goal of identifying a high-affinity ligand for the monoclonal antibody 3-E7 (Meo et al. 1983). Isolation of 3-E7 ligands is a well-defined in vitro selection problem characterized previously (Cwirla et al. 1990; Barrett et al. 1992). We designed our library to contain at least one known 3-E7 ligand, [Leu]enkephalin. The [Leu]enkephalin peptide binds to 3-E7 with an affinity of 7.1 nM, and its size (five residues) was well-suited for our experiments.

**Figure 7:**
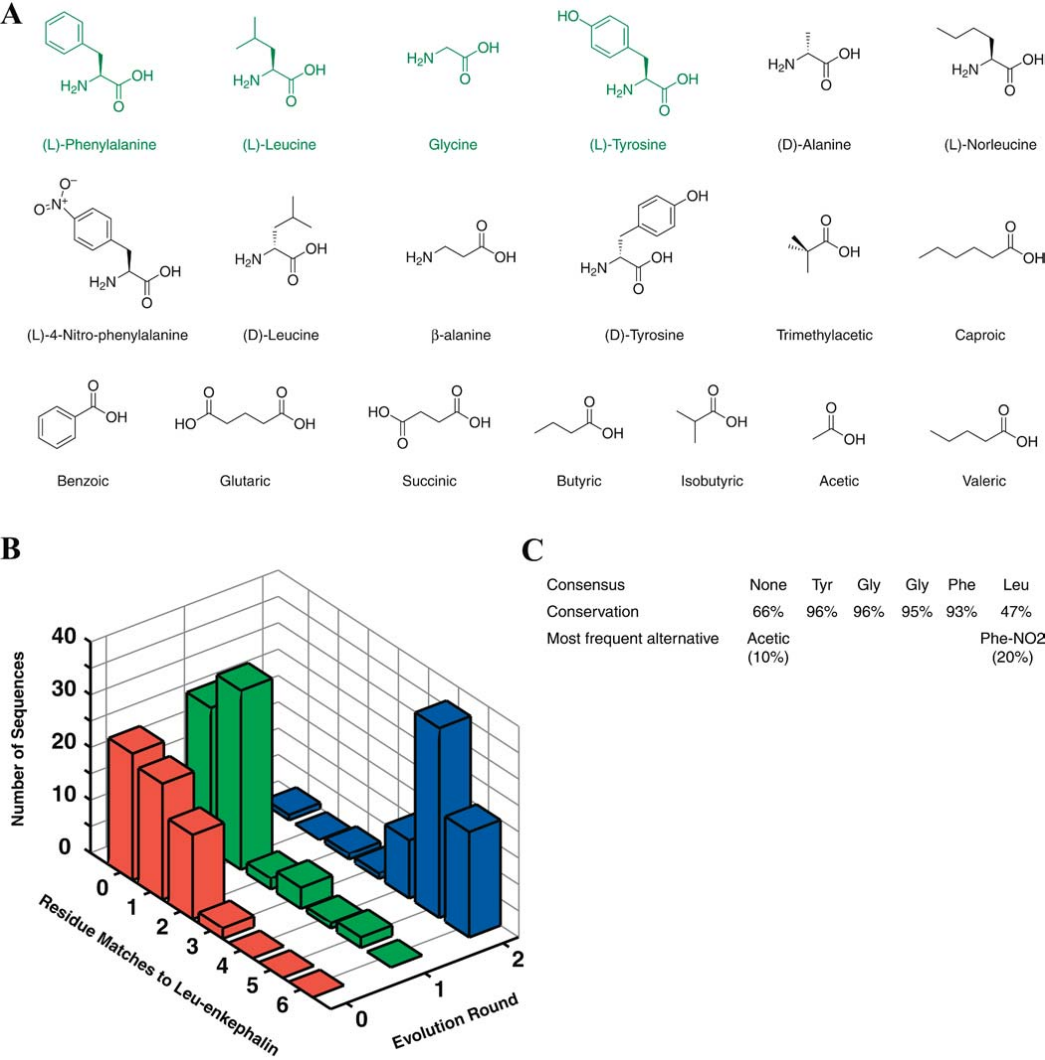
In Vitro Selection of a Nonnatural Peptide Library (A) Library building blocks. Proteinogenic building blocks are shown in green. (B) Approximately 70 DNA genes from each round of selection were sequenced, and the results are summarized as a histogram plot. The x-axis indicates the number of amino acid residue matches to [Leu]enkephalin encoded by a library sequence. The y-axis indicates the library generation (0, starting material; 1, after round one selection; 2, after round two selection). The z-axis indicates the number of sequences encoding a particular number of matches (x-axis) in a particular round (y-axis). (C) The top row reports the round two library consensus sequence, which matches [Leu]enkephalin. The second row reports the percentage of round two library clones that encode the [Leu]enkephalin amino acid at each residue position. The third row reports the identity and frequency of the most commonly occurring non-[Leu]enkephalin subunit at each position.

An initial DNA support library consisting of ten distinct sequences (“all a,” “all b,” etc.) was diversified 10^5^-fold by PCR recombination to generate a support library with a complexity of one million, as verified by DNA sequencing (Halpin and Harbury 2004). This library was chemically translated into acylated pentapeptides using Fmoc-protected succinimidyl esters. The peptide library included ten different monomers at each position (Figure 7A). The first five positions comprised one of ten amino acids (β-alanine, D-alanine, D-leucine, D-tyrosine, 4-nitro-phenylalanine, glycine, leucine, norleucine, phenylalanine, or tyrosine). The N-terminus was left unmodified or was acylated with one of nine acids (acetic, benzoic, butyric, caproic, glutaric, isobutyric, succinic, trimethylacetic, or valeric). After library synthesis and conversion of the ssDNA into duplex form, the library was subjected to selection using the 3-E7 antibody. The selected DNA was PCR amplified and used as input for the subsequent round of synthesis and selection.

In order to monitor library convergence, DNA from the starting material (round 0) and from after one (round 1) or two (round 2) selection generations was subcloned, and approximately 70 different isolates from each round were sequenced (Figure 7B). In round 0, none of the sequences encoded more than three residues in common with [Leu]enkephalin. Two sequences from round 1 encoded five [Leu]enkephalin residues, and one sequence encoded four residues. Of the round 2 sequences, twenty encoded full-length [Leu]enkephalin, thirty-four encoded single mutants, and eleven encoded double mutants. Only three round 2 sequences encoded less than four [Leu]enkephalin residues. The round 2 consensus peptide sequence matched [Leu]enkephalin (Figure 7C). Previous work has shown that the N-terminal residues (Tyr-Gly-Gly-Phe) are responsible for most of [Leu]enkephalin's affinity for the 3-E7 antibody (Meo et al. 1983; Cwirla et al. 1990). We observed high sequence conservation at these residues, recapitulating the earlier results.

To assess generality, we carried out a second [Leu]enkephalin in vitro selection experiment using a peptide library of the same size but constructed with a completely different “genetic code.” Every codon in the alternate library coded for an amino acid different from the one it coded for in the first library. The [Leu]enkephalin codon series in the first library was b_1_-j_2_-b_3_-c_4_-h_5_-i_6_, whereas in the alternate library it was d_1_-b_2_-g_3_-g_4_-i_5_-f_6_. Two rounds of selection enriched the alternate [Leu]enkephalin DNA gene 10^5^-fold (data not shown). The data suggest that little, if any, DNA sequence encoding bias exists in our system. Further, they illustrate the reproducibility of the technology. Together, the results demonstrate conclusively that the DNA display strategy can be used for the in vitro selection of synthetic chemical libraries.

## Discussion

Previous efforts to expand the scope of in vitro selection have utilized nonnatural bases or amino acids incorporated into DNA, RNA, and peptide libraries using polymerases and the ribosome (Bittker et al. 2002; Li et al. 2002; Forster et al. 2003). Such efforts are limited by the extent to which enzymes will tolerate novel monomers. In addition, enzymes can only produce polymers chemically and topologically similar to their natural products, which are not well-suited for all applications.

An alternative strategy for expanding the chemical diversity of gene products exploits DNA-templated synthesis, where hybridization-induced proximity promotes covalent bond formation (Gartner and Liu 2001). One great advantage of proximity-based DNA-directed synthesis is its ability to accommodate multiple reactions in “one pot.” However, there are several significant disadvantages. Each building block must be attached to an oligonucleotide, which is both expensive and labor intensive. All chemistry must proceed under conditions compatible with DNA hybridization, ruling out many organic solvents, high pH, and high temperature. Finally, there may be a limitation to the number of steps that can be encoded by the proximity approach. While an impressive array of chemical reactions has been accomplished by this method (Gartner et al. 2002), its use for in vitro selection has not been reported.

DNA display is a general method for the in vitro selection of synthetic combinatorial chemistry libraries. The system is modular, so that chemistry and selection protocols can be easily changed. It can take advantage of existing combinatorial chemistry technology as well as chemical transformations previously carried out in the presence of unprotected DNA (Gartner et al. 2002; Summerer and Marx 2002). Solid-phase, solution-phase, enzymatic, and proximity effect reaction formats are all suitable. We have developed an extensive set of tools to adapt new chemistries for in vitro selection (Halpin et al. 2004).

In addition to diverse chemistries, many different library architectures are also possible. The library reported here was synthesized in six encoded steps with ten distinct building blocks per step. However, essentially any combinatorial scheme can be accommodated. The 20-base codon sequences used here were taken from a larger set (>10,000) of 20-base sequences experimentally verified to exhibit orthogonal hybridization properties (Giaever et al. 2002).

As a first approximation, the highest possible fold enrichment per round of selection can be determined by considering its relationship to translation fidelity and the signal-to-noise ratio of the selection. Fold enrichment *(E)* is defined as the geometric increase in the fraction of target molecules in a library that results from a single round of synthesis and selection. Fidelity *(F)* is defined as the fraction of genes recovered from a completed library synthesis that have been correctly translated. The signal-to-noise characteristic of a selection *(S/N)* is defined as the ratio of the fraction of target molecules selected to the fraction of nontarget molecules selected. In most cases, the fold enrichment reduces to the simple expression at the right of Equation 1



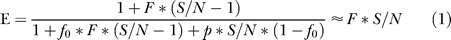



where *f_0_* denotes target gene fraction in the selection input and *p* denotes the probability of a nontarget gene being mistranslated to the target gene product. Biological systems have such high fidelity that *F* can be considered to equal one. However, the fidelity of chemical translation processes is the product of hybridization specificity and chemistry efficiency raised to the power of the number of steps. It is important to consider these parameters when adapting new chemistries and selections to the DNA display format. Equation 1 can help determine the minimum number of rounds required for library convergence, and thus the feasibility of a proposed in vitro selection experiment. For example, a library synthesized with a fidelity of 0.01 and subjected to a selection with a *S/N* of 1000 would give a 10-fold enrichment per round at best. If the library included 10^12^ unique members, at least 12 rounds would be required to achieve convergence.

In addition to influencing convergence rates, fidelity also limits achievable library complexity. The maximum effective library complexity corresponds to the product of Avagadro's number, the moles of library, and the fidelity. Based on our observed 90% hybridization efficiency and 95% chemistry efficiency, extension of the library reported here to 13 synthetic steps would produce 10^12^ distinct small molecules per 30 pmol of DNA starting material, a quantity easily manipulated in a microcentrifuge tube.

Diversification between rounds of selection by recombination makes possible in vitro evolution of libraries with complexities exceeding the physical library size. Thus, a “best” molecule can be pinpointed without exhaustive testing of all potential species. Starting with a working population of compounds that sparsely sample a chemical space, molecules containing parts of an optimal molecular solution often have a selective advantage relative to siblings, and become enriched. Subsequent recombination processes splice together fragments from the numerous partially optimal molecules to form a globally optimal molecule. Thus, the best structure is found, even if the odds were negligible that it existed in the initial working population. The same principle accounts for the striking success of gene shuffling in protein engineering (Kurtzman et al. 2001) and of the genetic algorithm optimization procedure in computer science (Forrest 1993). Recombination of a DNA display library by DNA shuffling (Stemmer 1994), which was used here to diversify the initial DNA library (Halpin and Harbury 2004), would enable the in vitro evolution of synthetic libraries with complexities exceeding 10^13^.

### Prospectus

DNA display enables the use of genetic tools such as complementation analysis and backcrossing to analyze small-molecule populations. The approach can be used to study molecular evolution without potential biases resulting from experiments restricted to RNA, DNA, and peptide polymers. A general scientific problem that will be directly addressed is the relationship between small-molecule library complexity and the quality of molecules discovered. With biopolymers, more complex libraries yield higher-affinity ligands (Takahashi et al. 2003). However, many have argued that increasing small-molecule library complexity will not produce higher quality “hits” (Breinbauer et al. 2002). This judgment is based on the paucity of viable drug candidates that have emerged from even the most complex combinatorial chemistry libraries. Analysis of “hits” from increasingly diverse small-molecule populations (as much as 10^6^-fold more complex than current synthetic libraries) will test the validity of this belief.

Drug discovery would represent one important application for a small-molecule in vitro selection technology. While the cost of drug discovery has increased continuously over the last decade (from less than $15 billion for research and development in 1996 to more than $25 billion in 2002), the number of new molecular entities approved by the FDA has steadily dropped, from 56 in 1996 to 17 in 2002 (Hall 2003). A fast, inexpensive, and generally accessible procedure for the in vitro selection of druggable small-molecule libraries would accelerate the early stages of drug development. The nonnatural peptide chemistry in this work was developed as a proof of principle, but may nevertheless have practical applications in medicine. For example, the nonribosomal peptide drugs vancomycin and cyclosporin are a widely used antibiotic and immunosuppressant, respectively (Walsh 2002). Annual joint sales of the nonnatural gonadotropin-releasing-hormone peptide analogues gosarelin and leuprolide exceed $2 billion (Klabunde and Hessler 2002). DNA display offers an immediate approach for the in vitro selection of general polyamide libraries that include such compounds (Halpin et al. 2004).

Future extensions of DNA display include the development of massively parallel array-based splitting strategies for the in vitro selection of low-molecular-weight small-molecule libraries (for example a library built in three synthetic steps with 10,000 building blocks per step). Massively parallel syntheses will produce compounds that conform better to Lipinski's “rule of five” (Lipinski et al. 1997) and presumably will thus be more druggable. Beyond drug discovery, DNA display can be applied to the engineering of chemical switches, the discovery of transition metal catalysts for aqueous and nonbiological environments, and the identification of enzyme-specific ligands for activity-based profiling. Because the system is inexpensive, is easily implemented by a single individual, and requires only common laboratory equipment, in vitro selection and eventual evolution of large synthetic chemical populations should become a broadly accessible tool.

## Materials and Methods

### 

#### Materials.

The 3-E7 antibody was purchased from Gramsch Laboratories (Schwabhausen, Germany). PANSORBIN cells were purchased from Calbiochem (San Diego, California, United States). BSA was purchased from New England Biolabs (Beverly, Massachusetts, United States). Yeast tRNA was purchased from Ambion (#7119, Austin, Texas, United States). The [Leu]enkephalin peptide and all oligonucleotides were purchased from the Stanford PAN Facility (Stanford, California, United States).

#### Chemistry.

Solid-phase peptide synthesis was carried out as previously described (Halpin et al. 2004). 5′ amino-modified ssDNA (#10-1912-90, #10-1905-90, #10-1918-90, Glen Research, Sterling, Virginia, United States) was noncovalently bound to DEAE Sepharose Fast Flow resin (# 17-0709-01, Pharmacia-LKB Technology, Uppsala, Sweden) packed into TWIST column housings (Glen Research #20-0030-00). DNA was loaded onto the columns in 10 mM acetic acid, 0.005% Triton X-100 buffer. To accomplish amino acid additions, columns were washed with 3 ml of DMF and subsequently incubated with 62.5 mg/ml Fmoc succinimidyl esters in 300 μl of coupling solvent (22.5% water, 2.5% DIEA, and 75% DMF) for 5 min. Excess reagent was washed away with 3 ml DMF, and the coupling procedure was repeated. The Fmoc-protecting group was then removed by two 1-ml treatments with 20% piperdine in DMF, one for 3 min and one for 17 min (Carpino and Han 1970). Finally, the columns were washed with 3 ml of DMF followed by 3 ml of DEAE Bind Buffer (10 mM acetic acid, 0.005% Triton X-100). Anhydride couplings followed the same procedure except that a 3-ml water wash was added after DNA loading to remove remaining acetic acid. Columns were incubated with 10 mM of each anhydride (100 mM for trimethylacetic anhydride) in 500 μl of DMF for 30 min. 20-base oligonucleotide–peptide conjugates were eluted off DEAE columns with 2 ml of DEAE Elute Buffer (1.5 M NaCl, 50 mM Tris pH 8.0, and 0.005% Triton X-100). 340-base ssDNA-peptide conjugates were eluted with 2 ml of Basic Elute Buffer (1.5 M NaCl, 10 mM NaOH, and 0.005% Triton X-100) heated to 80 °C. For synthesis of libraries, a 2-ml PBS wash was added at the end of each amino acid coupling step to remove remaining anionic reagents. Following the last coupling step in the library synthesis, free oligonucleotides were separated from 340-base DNA supports by washing with 2 ml of DEAE Elute Buffer.

#### Electromobility shift assay.

The electromobility shift assay was performed as previously described (Hwang et al. 1999). No plasmid DNA was added to the samples. Antibody 3-E7 (0.5 μg) was added to the “antibody plus” samples. Samples were run on a 2% NuSieve (#50081, FMC Bioproducts, Rockland, Maryland, United States) agarose gel for 1 h at 100 V in TBE. 840 μM peptide was used to compete away binding to the peptide–DNA conjugate.

#### Selection.

ssDNA was converted to double-stranded DNA (dsDNA) by one-cycle PCR with a single end primer. The 50-μl PCR reaction contained 20 μM primer, 200 μM of each dNTP, 5 mM MgCl_2_, 1X Promega Taq reaction buffer, and 5 U of Taq DNA polymerase (#M1661, Promega, Madison, Wisconsin, United States). The PCR program was 94 °C for 2.5 min, 58 °C for 1 min, and 72 °C for 15 min. The dsDNA–peptide conjugates were incubated with PANSORBIN cells in 50 μl of Selection Buffer (TBS, 0.1% BSA, and 0.1 μg/μl yeast tRNA) at 4 °C for 1 h to preclear conjugates that nonspecifically bind to the cells. Then, preclear beads were pelleted by centrifugation and removed. Antibody 3-E7 (0.5 μg) was added to the supernatant and allowed to incubate for 1 h at 4 °C. The solution was then mixed with fresh PANSORBIN cells for 1 h at 4 °C. The cells were pelleted and washed at 25 °C three times with 500 μl of Wash Buffer (TBS, 0.1% BSA, 0.1 μg/μl tRNA, and 350 mM NaCl), followed by a single wash with 500 μl of Selection Buffer. The dsDNA–peptide conjugates were eluted by incubation of the cells with 50 μl of 200 μM [Leu]enkephalin in Selection Buffer for 1 h at 25 °C. Selected genes were amplified from 10 μl of elute supernatant with 25-cycle PCR reactions.

#### General.

High performance liquid chromatography analysis of DNA–peptide conjugates, synthesis of anticodon columns, hybridization and transfer of DNA, library assembly, ssDNA generation, and library isolation were performed as previously described (Halpin and Harbury 2004; Halpin et al. 2004).

## Abbreviations

DEAE: diethylaminoethyl
DMF: *N,N*-dimethylformamide
dsDNA: double-stranded DNA
Fmoc: 9-Fluorenylmethoxycarbonyl
HTS-CC: high-throughput screening of combinatorial chemistry
ssDNA: single-stranded DNA
